# PD130 Health Technology Assessment Of Cervical Cancer Screening In Indonesia

**DOI:** 10.1017/S0266462324003672

**Published:** 2025-01-07

**Authors:** Suzanna Mongan, Joshua Byrnes, Alfred Lam, Hansoo Kim

## Abstract

**Introduction:**

Cervical cancer is the most common gynecological cancer and the second most common cancer among women in Indonesia, and its incidence is projected to increase. In 2018, the mortality rate of cervical cancer was 60 percent, since patients often have advanced stage disease at diagnosis. We aimed to identify the status and cost of cervical cancer screening in Indonesia.

**Methods:**

We conducted a literature review with search terms relevant to cervical cancer, screening, and Indonesia in two major databases—Embase and Medline—and by citation searching through the World Health Organization (WHO) website, human papillomavirus (HPV) reports, and Indonesian reports on cervical cancer.

**Results:**

Indonesia’s national cervical cancer screening program began in 2007 and uses visual inspection with acetic acid (VIA) or the Papanicolaou (Pap) test. VIA has high sensitivity (94%) and specificity (95%). It is the preferred option due to the limited number of pathologists and its lower cost (IDR25,000 [USD1.64]), compared with the Pap test (IDR200,000 [USD13.13]) and the HPV DNA test (IDR550,000 [USD36.00] to IDR1,400,000 [USD 91.90]). VIA and the Pap test are covered by national health insurance, whereas the HPV test is not. Nevertheless, national screening coverage was less than 10 percent, with a wide regional disparity due to the limited number of screening providers, resource constraints, and lack of awareness.

**Conclusions:**

In Indonesia, VIA is the primary screening method because of its affordability, accessibility, and applicability in low-resource settings. However, low screening coverage has led to a high incidence of cervical cancer. New policies and incentives are needed to ensure adequate numbers of screening providers and monitoring systems. Health technology assessment can help choose cost-effective strategies for primary HPV DNA testing.

